# Tinzaparin—a review of its molecular profile, pharmacology, special properties, and clinical uses

**DOI:** 10.1007/s00228-022-03365-4

**Published:** 2022-07-23

**Authors:** Marina Amerali, Marianna Politou

**Affiliations:** Hematology Laboratory-Blood Bank ARETAIEION Hospital, School of Medicine, National and Kapodistrian University of Athens, Vasilissis Sofias 76, 11526 Athens, Greece

**Keywords:** Thrombosis, Heparin, Low molecular weight heparin, Tinzaparin, Obesity, Elders, Oncology, COVID-19

## Abstract

**Purpose:**

Low molecular weight heparins (LMWHs) are a group of heterogenous moieties, long used in the prevention and treatment of thrombosis. They derive from heparin and since they are prepared by different methods of depolymerization, they differ in pharmacokinetic properties and anticoagulant profiles, and thus are not clinically interchangeable.

**Methods:**

In this review we provide an overview of tinzaparin's main characteristics and uses.

**Results:**

Tinzaparin which is produced by the enzymatic depolymerization of unfractionated heparin (UFH) can be used for the treatment and prevention of deep venous thrombosis (DVT) and pulmonary embolism (PE); it has been also used in special populations such as elders, obese, pregnant women, and patients with renal impairment and/or cancer with favorable outcomes in both safety and efficacy, with a once daily dose regimen. Furthermore, LMWHs are extensively used in clinical practice for both thromboprophylaxis and thrombosis treatment of COVID-19 patients.

**Conclusion:**

Tinzaparin features support the hypothesis for having a role in immunothrombosis treatment (i.e. in the context of cancer ,COVID-19), interfering not only with coagulation cascade but also exhibiting anti-inflammatory potency.

## Introduction

Tinzaparin sodium belongs to the family of heparinoids and more specifically to low molecular weight heparins (LMWHs). LMWHs are a group of heterogenous mixtures of oligosaccharides deriving from unfractionated heparin (UFH), produced by depolymerization. The method of depolymerization (chemical cleavage with different agents such as nitrous acid, isoamyl nitrate, alkaline treatment, or enzymatic treatment with heparinase) gives each agent specific chemical and pharmacological characteristics, resulting in differences in molecular weights (MWs), bioavailability, and indications for use [1].

Heparin is structurally like endogenous heparan sulfate (HS), which is involved in various biological procedures such as thrombosis, angiogenesis, inflammation, and tumor metastasis. That similarity concedes to UFH anti-inflammatory and anti-oncogenic properties [2].

Hemostasis, the process that leads to cessation of bleeding, involves a series of clotting factors’ activation (coagulation cascade) ultimately leading to the polymerization of fibrin and the formation of a clot with platelets and fibrin polymers. Extensive activation of this process leads to thrombosis, highlighting the importance of interim equipoise on the activation/inactivation of the cascade [3]. Very recently, close interactions between innate immunity, inflammation, and coagulation have also been described. The process, which is called immunothrombosis is an innate immune response in which the local activation of blood coagulation exerts a protective effect against microbes or trauma. Neutrophils recruitment and activation with subsequent NETosis (release of neutrophil extracellular traps), endothelial cell damage and activation, platelet activation and aggregation, and platelet direct interactions with innate immune cells (i.e., neutrophils) or secretion of cytokines/chemokines together with coagulation protease activation, all participate in the complex process of immunothrombosis. The key role of immunothrombosis in pathologic states including thrombosis, cancer, sepsis, and trauma has been also recognized [4].

LMWHs have been used in clinical practice for about half a century, since 1980s. Their main administration route is subcutaneously, and they are used both for prophylaxis and treatment of thrombosis [5]. The main indications of LMWHs are treatment of deep venous thrombosis (DVT) with or without pulmonary embolism (PE) and prophylaxis in patients undergoing surgery, coronary syndromes, and hemodialysis. Unlike UFH, their main elimination route is renal and as they convey a lower affinity for plasma proteins, demonstrating a more predictable bioavailability profile [6]. Tinzaparin sodium is a LMWH, produced by depolymerization via heparinase, an enzyme derived from *Flavobacterium heparinum*. It is available on the market in several forms of prefilled syringes and multi-dose vials for once daily administration according to product’s monograph, thus making the use more convenient. It is recommended for the treatment of DVT with or without PE, for extended treatment of venous thromboembolism and prevention of recurrences in adult patients with active cancer but also for VTE prophylaxis for both non-surgical immobilized patients (due to acute heart failure, acute respiratory failure, severe infections, active cancer, as well as exacerbation of rheumatic diseases) as well as in adult patients undergoing surgery, particularly orthopedic, general, or oncological surgery. It is also indicated for prevention of clotting in extracorporeal circuits during hemodialysis and hemofiltration in adults [6, 7] and in our knowledge, tinzaparin is widely used in various European countries, especially for the management of cancer-associated thrombosis (CAT) [8–10].

The main characteristics of UFH and LMWHs, tinzaparin, enoxaparin, and dalteparin, are summarized in Table 1 and further analyzed in the main part of this review.

**Table 1:** Main characteristics of tinzaparin, enoxaparin, dalteparin, UFH

**Characteristic**	**UFH**	**LMWHs**		
		**Tinzaparin**	**Enoxaparin**	**Dalteparin**
Average MW (Daltons-Da) [7, 11]	5000–30000 Da	6500 Da	4500 Da	6000 Da
Metabolism [1–4, 12]	Liver Kidneys	Kidneys^a^ Reticuloendothelial system (RES)	Kidneys^a^	Kidneys^a^
Elimination half-life [1–4, 12]	Dependent on the dose	3–4 h	5 h (single dose)	3–4 h
Renal accumulation [1–4, 12]	No	No	Yes	No (prophylaxis doses) Yes (treatment doses)
Anti-Xa/anti-IIa activity ratio [13–16]	1	1.9	3.6	2.5
Monitoring	+ (aPTT)	- (Anti-Xa levels, not routinely needed)	- (Anti-Xa levels, not routinely needed)	- (Anti-Xa levels, not routinely needed)
Antidote [7]	Protamine sulfate	Protamine sulfate^a^ (anti-Xa neutralization 85.7%)	Protamine sulfate^a^ (anti-Xa neutralization 54.2%)	Protamine sulfate^a^ (anti-Xa neutralization 74.0%)
Dose Regimen [1–4]	IV (mainly), SC Continuous infusion	SC Once daily	SC Once daily/twice daily	SC Once daily/twice daily
Preparation [17, 18]	-	Enzymatic depolymerization via heparinase (*Flavobacterium heparinum*)	Chemical depolymerization OR chemical cleavage—alkaline treatment	Chemical depolymerization OR chemical cleavage—nitrous acid

^a^Further analysis in Discussion

**Fig. 1:**
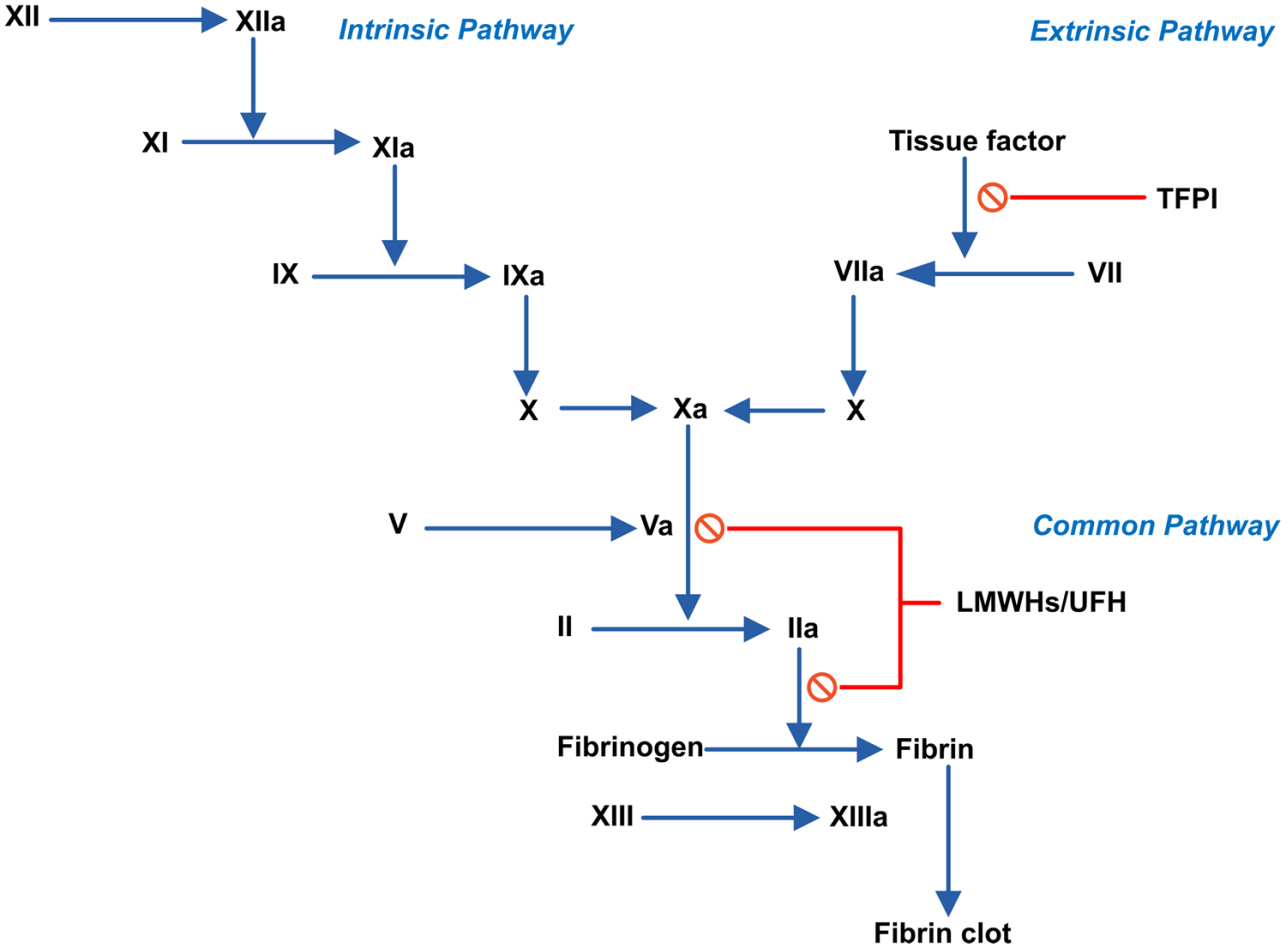
A simplified depiction of coagulation cascade and LMWHs sites of action

LMWHs interfere with the coagulation cascade (see Fig. 1) by enhancing the inhibitory effect of Antithrombin III (ATIII) mainly on activated factor X (FXa) and thrombin (FIIa), but also on activated FIX, and activated FXII by several orders of magnitude. LMWHs also influence the regulation of the tissue factor (TF) pathway by releasing the tissue factor pathway inhibitor (TFPI) from the endothelium, but also in part by inhibiting the generation and activity of FVIIa in an AT-dependent manner [19]. Differences in molecular weight result in differences in ATIII binding affinity affecting the subsequent half-life, anti-Xa, and anti-IIa activities of LMWHs. Furthermore, the release of endothelial tissue factor pathway inhibitor (TFPI) is directly dependent on the molecular weight and the degree of sulfation. Thus, different LMWHs demonstrate different capacity of releasing endothelial TFPI [20].

The aforementioned differences in PD profiles do not allow the interchangeable clinical use of LMWH [1, 21].

### PK/PD

Enzymatic preparation of tinzaparin offers some advantages in its chemical composition. Being a “more natural” method, it is thought to cause less damage to the molecules while a high degree of sulfation of the chains is retained. This compound contains a range of short and long chains, with an average molecular weight (MW) of 6500 Da, greater than all other LMWHs available. The proportion of ultra-short chains of < 2000 Da does not exceed 10% [6, 22]. Barrett et al. conducted a study with tinzaparin and a tinzaparin-like agent with a higher proportion of short chains to estimate the pharmacodynamic changes and the importance of low molecular weight chains’ presence, without finding significant differences in anti-Xa and anti-IIa activities in vivo [21]. The longer chain lengths of tinzaparin seem to result in greater inhibition of factor IIa compared to LMWHs with shorter chains. Binding of LMWHs to AT depends on a unique, highly sulfated, five-residue sequence found in approximately 30% of heparin molecules. A longer average length of the chains in a LMWH preparation increases the probability that they will contain this pentasaccharide sequence, allowing them to exert the AT-dependent effects. The chains must consist of at least 18 saccharide units to have an anti-IIa activity. Molecules in a LMWH preparation that are less than 18 saccharide units in length are still able to inactivate factor Xa. However, they are too short to form the ternary complex that is required to inactivate thrombin. The combination of partial inactivation of thrombin (factor IIa, by the longer chains in the mixture) and the inactivation of factor Xa (an essential component in the formation of new thrombin) provide to tinzaparin an adequate anticoagulant effect [13].

In the past, the activity of LMWHs was sometimes expressed as an anti-Xa/anti-IIa ratio. Since tinzaparin has a higher anti-IIa activity, this is reflected in a lower anti-Xa/anti-IIa ratio. The anti-Xa/anti-IIa ratio of tinzaparin is 1.9, a ratio closer compared to other LMWHs to that of UFH (anti-Xa/anti-IIa ratio of UFH is 1.0) demonstrating a similar anti-Xa activity with other LMWHs but a higher anti-IIa activity [5, 7, 19].

TFPI represents an alternative natural anticoagulant mechanism (separate from the AT mechanism). The main function of TFPI is to eliminate from the clotting cascade the factor VIIa-tissue factor (FVIIa-TF) complex (a complex formed early in the coagulation cascade, after endothelium damage). It exerts its effect through a factor Xa-dependent mechanism: TFPI forms a complex with Xa, thereby inhibiting Xa activity. The TFPI-FXa complex then binds to the VIIa-TF complex on the endothelial cell membrane surface and blocks its activity. In addition to its anticoagulant role, TFPI, also has other non-anticoagulant roles; e.g., it may have beneficial effects in reducing sepsis, inflammation, and angiogenesis [23–28].

LMWHs stimulate release of TFPI from endothelium, a function depending on the chain length, with fractions with a molecular weight of > 6–8 kDa stimulating the highest release. Additionally, a high degree of sulfation of the chains also appears to contribute to the release of TFPI [20]. Tinzaparin has been shown to cause a greater TFPI release compared to bemiparin, a property which has been attributed to its larger mean molecular weight and the higher sulfatation level of its chains [29]. Also, patients treated with long-term (90 days) tinzaparin had 2–2.5-fold increased TFPI levels throughout their treatment period while TFPI levels of patients treated with UFH dropped significantly after 20 days of treatment [28].

Due to the higher proportion of long chains and high sulfate content, which correlates with high reversal of anticoagulation effect, an efficient neutralization via the protamine sulfate is achieved for tinzaparin, at about 85.7% for anti-Xa activity [7].

LMWHs’ clearance depends also on their chain length and molecular weight which affect the potency of binding to both ATIII and endothelial cells. In early studies LMWHs were thought to be eliminated by the kidneys in a so-called non-saturable way. However, new studies have demonstrated a relationship between MW and elimination route [30, 31]. The broader distribution of heparin chain lengths in tinzaparin leads to a higher affinity for plasma proteins and thus to an elimination which is less dependent on the kidneys and is performed via the reticuloendothelial system (RES), offering a more favorable profile for patients with renal impairment [31, 32].

Unlike UFH, whose activity and dosing need a continuous monitoring via aPTT measurements, LMWHs seem to lack such a need because of their more predictable bioavailability and safety. Although anti-Xa IU/ml concentration in plasma constitutes an acceptable marker for the indirect estimation of LMWHs’ activity, since LMWHs are mixtures of polysaccharides that cannot be assessed directly in plasma, anti-Xa plasma levels is not definitively related to the clinical anticoagulant effect of LMWH and thus, its measurement is not routinely advised in clinical practice except in specific populations [33].

The standardized dose of LMWHs is expressed in anti-Xa International Units (IU). Tinzaparin’s recommended treatment dose is 175 IU/kg subcutaneously (SC) once daily, according to agent’s summary of product characteristics (SmPC). The lack of need for monitoring, the administration route, pharmacokinetics and pharmacodynamics, and the option for use in outpatient basis render LMWHs advantageous over UFH [34]. In addition, the risk for heparin-induced thrombocytopenia (HIT) is lower with these agents [35]. Heparin-induced thrombocytopenia (HIT) is an immune-mediated disorder caused by antibodies that recognize complexes of platelet factor 4 (PF4) and heparin. Thrombosis is a central and unpredictable feature of this syndrome; HIT occurs in 0.5 to 1% of patients exposed to unfractionated heparin for medical and surgical indications. The incidence is markedly lower (0.1–0.5%) in patients receiving LMWH.

Tinzaparin’s absolute bioavailability based on anti-Xa activity after subcutaneous administration is approximately 90% and the time to reach maximal activity is 4–6 h. The terminal elimination half-life is approximately 3.7 h. Due to the long half-life of the pharmacological effect for tinzaparin, once daily administration is sufficient.

The anti-Xa profile of tinzaparin supports the pharmacodynamic superiority of low molecular weight heparins over standard intravenous (IV) heparin administration. The latter demonstrates a bioavailability of about 30%, attributed to its binding to plasma proteins and intracellular degradation, with a great extent of inter-individual variability [6].

The elimination half-life of tinzaparin is estimated at about 1.5 h for anti-Xa and 1.25 h for anti-IIa after SC administration, with no residual anti-Xa activity occurring after 24 h, consequently allowing for once daily dose [6].

## Once vs twice daily administration

Once daily administration of LMWHs is preferable for patients, especially those with cancer and elders, since it causes less discomfort [36, 37]. Nonetheless the equilibrium between safety and efficacy must always be balanced since concerns of inefficiency in one-dose schemes have arisen. In a Cochrane database systematic review by Bhutia et al., the safety and efficacy of the administration of different LMWHs once or twice daily were studied and it was found that there was no significant difference for either recurrence of thromboembolism or major bleedings [36].

There is a controversy in regard of enoxaparin, with studies showing no difference between once and twice daily dose [38], or even a better safety profile (with fewer major bleeds and deaths in patients with the once daily regimens while others demonstrated a lower efficacy for once daily scheme) [39]. In terms of tinzaparin, an early study of Siegbahn et al. demonstrated no significant difference between once and twice daily dose neither in efficacy nor safety [40], a finding that was confirmed by a retrospective analysis of Nelson-Piercy et al., in pregnant women [41].

## Specific populations

It is worth mentioning that most PK studies are conducted in healthy volunteers and despite the predictable anticoagulant potential described above, a concern for special populations such as pregnant women, elders, and patients with renal impairment remains. Physiologic changes in these populations lead to differences in pharmacokinetics and pharmacodynamics. Increased or decreased plasma volume, the fluctuation of glomerular filtration rate (GFR), and the presence of placental heparinase lead to re-estimation of dosing and pharmacologic profile of the drug in these populations [33].

### Pregnancy

Pregnant women are at high risk for thrombosis, with VTE and PE being important causes of maternal morbidity and mortality and LMWHs are the preferred agent for treatment of thrombosis in pregnancy [42]. The majority of the studies demonstrate a favorable profile for tinzaparin [41–44], and monitoring Xa activity may be an attractive option especially for long-term treatment in this population [45, 46]. No neonatal adverse effects related to tinzaparin were described [45]. LMWH preparations contain benzyl alcohol, as a preservative, which may cause toxic and anaphylactoid reactions in infants, but prefilled syringes of tinzaparin do not contain benzyl alcohol and therefore can be used during pregnancy. Tinzaparin seems to be well tolerated in pregnancy; thus, larger studies are needed to confirm the aforementioned results.

### Obesity

Obese patients are at higher risk for VTE. According to Barrett et al., tinzaparin dose adjusted to body weight is preferable for all individuals, thus anti-Xa activity is not related to body weight [21]. According to Hainer et al., tinzaparin dose should be adjusted to body weight even in overweight patients, since there is no maximum permissible daily dose [47]. Moreover, it has been speculated that tinzaparin may favor obese patients by lowering the cardiovascular risk through the reduction of the levels of common inflammatory markers such as von Willebrand factor (vWF) and TNF-a [48].

### Chronic kidney disease (CKD)-renal impairment (RI)

Patients with end-stage renal disease are at risk of developing thrombosis due to increased levels of vWF, fibrinogen, and lipoprotein(a). Patients undergoing hemodialysis may have additional prothrombotic risk factors such as catheter placements and erythropoietin therapy. The equilibrium between thrombotic complications and bleeding is fragile, requiring an antithrombotic agent with both efficacy and safety, with tinzaparin offering several advantages [32]. As mentioned above, LMWHs are mainly eliminated by the kidneys, with a fluctuating elimination rate depending on their MW [30, 49]. Tinzaparin’s clearance is less dependent on renal elimination route [31, 32]. In a study of Hainer et al., tinzaparin at the fixed dose of 75 IU/kg SC as prophylaxis on the off-dialysis day and intravenous (IV) on the day of hemodialysis session was administered to patients with adequate tolerance [50]. Tinzaparin pharmacokinetics seems not to be affected by renal impairment (RI) since anti-Xa activity measurement has not demonstrated tinzaparin’s accumulation in patients with mild to moderate renal insufficiency and creatinine clearance (CrCl) down to 20–30 ml/min as an estimate of GFR for up to 30 days of treatment [51–54]. In patients with a CrCl < 20 ml/min, the dose can be adjusted based on anti-Xa level measurement [51, 52, 55, 56].

In terms of safety, tinzaparin has shown similar bleeding rates in patients with and without renal insufficiency while long-term therapy in cancer patients with RI did not increase clinically relevant bleedings.

When compared to enoxaparin in patients with RI, tinzaparin has shown no statistically significant accumulation [56] while enoxaparin was associated with increased bleeding risk and a dose adjustment was recommended especially when GFR < 30 ml/min [57].

### Elders

Patients aged > 70 years old constitute a large proportion of patients in need for anticoagulants as the risk for VTE and atrial fibrillation increases with age [58]. The major concern for this group is renal impairment, analyzed above, as GFR decreases with age. Very old individuals (> 80 years old) are also prone to falls and bleeding disorders, due to their frailty.

According to Mahe et al., tinzaparin showed a more favorable pharmacodynamic profile in elders with renal impairment compared to enoxaparin [56]. Monitoring and dose-adjustment are not generally needed and advised [52] but may offer great advantages for the treatment of especially very elderly patients [55].

## Tinzaparin in oncology

Malignancies are strongly related with VTE, and apart from the tumor itself, many cofactors such as chemotherapy, immunotherapy [59] erythropoietin use, and steroids augment the risk for thrombosis compared to general population. There is a well-characterized interplay between coagulation and cancer since tumor promotes procoagulant agents and thrombin generation and the latter may promote tumor growth and metastases [60]. TF produced by several tumor cell types seems to play an important role in both coagulation and primary tumor growth and metastasis [61–65] contributing to the pathophysiology of cancer with either thrombin-dependent or independent mechanisms [66]. TF is primarily responsible for both tumor-induced thrombin generation (by direct activation of the coagulation pathway) and the formation of tumor cell-platelet aggregates [67]. TF bearing procoagulant microparticles can also contribute to that process [65, 68]. Besides the pivotal role of thrombin in thrombosis, it is traditionally acknowledged that many effects of thrombin in cancer may be independent of its clotting activity. Thrombin might contribute to cancer biology by activating platelet-tumor aggregation and promoting cellular proliferation, tumor adhesion to subendothelial matrix, or act through direct protease-activated receptor (PAR)–mediated cell signaling, leading to production of soluble cytokines and angiogenic growth factors interfering with tumor growth, tumor-associated angiogenesis, and metastasis [65, 69]. Because of the pivotal role of TF and thrombin generation in cancer growth and spread [70], it is conceivable that their inhibition could play a role not only in reducing the prothrombotic properties of the tumor but also affecting its growth and metastatic potential [63, 71].

LMWHs are used for the treatment and prophylaxis of VTE in cancer patients [72, 73]. Patients with cancer may also have comorbidities such as obesity, renal failure and they are usually of older age. Therefore, it is important to use an antithrombotic agent that can reduce the thrombotic risk while maintaining a low bleeding risk [74]

Due to its pharmacokinetics and pharmacodynamics, there is evidence supporting the use of tinzaparin in patients with active malignancies [75].

In the largest trial (ClinicalTrials.gov Identifier: NCT01130025) that has studied the efficacy and safety of full dose tinzaparin (175 IU/kg) daily compared to warfarin for the treatment of acute VTE in patients with active cancer, recurrent VTE occurred in 31 patients in the tinzaparin group and 45 patients in the warfarin group (cumulative risks, 7.2% for the tinzaparin group vs 10.5% for the warfarin group; hazard ratio [HR], 0.65 [95% CI, 0.41–1.03]; *P* = 0.07, while tinzaparin was associated with a lower rate of clinically relevant non major bleeding [76].

In a systematic review of Martinez et al., tinzaparin was found to be superior in the 12-monthsfollow-up in terms of VTE recurrence [77] suggesting that tinzaparin is also a safe option for extended long-term treatment [78]. Furthermore, tinzaparin seems to be superior to vitamin K antagonists (VKAs) for preventing both post-thrombotic syndrome and venous ulcers [79, 80].

Despite the many and various mechanisms involved in the multifaceted relationship between cancer and thrombosis, anticoagulants might represent an attractive therapy, as current research supports the hypothesis that such drugs might also offer a better control of cancer progression. In vitro studies have demonstrated an anti-oncogenic and an anti-metastatic effect of tinzaparin which have been attributed to (a) the TFPI, and its property of inhibiting both procoagulant and non-coagulant effects of TF [81], (b) its interference in the angiogenesis process which was shown to be dose-related and dependent on the relatively higher molecular weight tinzaparin fragments, and (c) its ability to prevent chemo-resistance in certain cancer types [81–84].

Metastases’ development may be reduced because of chemokine receptor 4 (CXCR4) signaling inhibition by LMWH. According to a study on Chinese hamsters, tinzaparin can inhibit CXCR4-SDF1 interaction by binding stromal cell–derived factor-1 (SDF-1). In that study, tinzaparin reduced the frequency of metastases of breast cancer [85]. The anti-metastatic effect of LMWH may depend on the inhibition of endothelial cell adhesion. Tinzaparin was confirmed to inhibit selectins more effectively than other LMWHs [86].

In an experimental model of human colon cancer, tinzaparin administration 24 h after angiogenesis stimulation by VEGF led to a decrease of the angiogenic index to the control level [87]. The effect of tinzaparin on lung metastases of melanoma B16 was also studied in a mouse model. A single, subcutaneous drug dose before the cancer cell inoculation reduced metastatic tumor formation by 89% in comparison to the control. Repeated tinzaparin administration once a day for 14 days before the cancer cell infusion caused a 96% reduction of the frequency of lung tumors [83]. In a recently published study, based on combinatorial therapy approaches to treat highly malignant and refractory cancers such as pancreatic cancer (PC), the authors hypothesized that tinzaparin can augment the effectiveness of traditional chemotherapeutic drugs and induce efficient antitumor activity. PANC-1 and MIAPaCa-2 cells were incubated alone or in combination with tinzaparin, nab-paclitaxel and gemcitabine. In vivo evaluation of these compounds was performed in a NOD/SCID mouse using a model injected with PANC-1. The triple regimen provided an extra 24.3% tumor reduction compared to the double combination (gemcitabine plus nab-paclitaxel) [88].

Whether such an in vitro effect is translated in progression free survival (PFS) was questioned in PaCT study [89] and the investigators demonstrated that the administration of tinzaparin in advanced pancreatic cancer (PaC) patients undergoing chemotherapy resulted in 39.5% higher PFS than in patients without such thromboprophylaxis.

## COVID-19

During the COVID pandemic era, it was soon observed that COVID patients were at a high risk for developing both arterial and venous thrombosis [90]. SARS-CoV infection causes a proinflammatory environment and immunothrombosis seems to play an important role in COVID-19 pathogenesis.

Although all guidelines recommend starting anticoagulation for venous thromboprophylaxis in all hospitalized patients with COVID-19, preferably with LMWH, they currently represent living guidance in view of the results of randomized clinical trials. Open questions remain regarding the choice of agent, the optimal dosing of anticoagulation based on illness severity, as well as the utility of VTE prophylaxis after hospital discharge. Based on the latest evidence, in moderately ill hospitalized COVID-19 patients on low flow oxygen, full dose anticoagulant prophylaxis with LMWH can be considered in patients with low bleeding risk, for 14 days or until discharge (whichever happens first), as this may improve patient survival until hospital discharge without the need for ICU-level organ support. Also, in critically ill hospitalized COVID-19 patients with no contra-indications to anticoagulation, prophylactic dose of anticoagulant is suggested over full treatment dose [91, 92].

Considering the key role of increased thrombin generation (factor IIa) and tissue factor (TF) pathway activation in COVID-19-associated thrombosis [93], the special features of tinzaparin (higher anti-IIa activity and TFPI release) support the hypothesis that tinzaparin may have an extended role, interfering not only with coagulation cascade but also exhibiting its anti-inflammatory potency when used for the thrombosis treatment and prophylaxis for COVID-19 patients. In the hypothesis of Belen-Apak, inhibition of FXa could lead to lower SARS-CoV viral load, as FXa plays a role in the viral entrance mechanism. Hence, LMWHs and especially tinzaparin and dalteparin are suggested for COVID-19 treatment [94].

The trial of Jonmarker et al. (ClinicalTrials.gov identifier NCT04412304) supported the use of high-dose tinzaparin or dalteparin for thromboprophylaxis for critically ill patients, showing a reduction of mortality without major bleeding events [95]. The study compared only different dosage schemes and not differences between agents.

Three case reports of middle-aged males with PE, as a complication of COVID-19, all treated with tinzaparin are also presented in the literature [96–98]. In the first two cases, tinzaparin was used as bridge-treatment in hospital and both patients received an oral anticoagulant to continue treatment after hospital discharge with good outcome [96, 97]. The third case is of great interest as he presented, with both arterial and venous thromboses, while being under thromboprophylaxis with nadroparin. After the first event (stroke) occurred the dose was increased but a couple of days later, he developed PE. A new regimen with tinzaparin led to a favorable outcome [98].

In the recently published INTERACT study, a higher than conventionally used prophylactic dose of anticoagulation with tinzaparin was administered for VTE prevention in 705 hospitalized, non-critically ill COVID-19 patients with moderate disease severity. The median duration of treatment was 13 days, reflecting the hospitalization period. In total, 14 thrombotic (2.0%) and four bleeding events were observed (0.6%) during the observation period. In-hospital death occurred in 12 patients (1.7%) due to disease progression. For the total cohort, laboratory parameters (d-dimers, CRP, and PLTs), and the SpO_2_ measurements showed significant improvements over time. For most patients, the WHO progression scale score dropped over time indicating health improvement [99]. The authors concluded that prophylactic anticoagulation with an intermediate to full therapeutic dose of tinzaparin among non-critically ill patients hospitalized with COVID-19 was safe and effective; tinzaparin might be superior to other anticoagulants in treatment and prophylaxis for COVID-19 patients but further studies are needed to confirm these results.

## Conclusion

Tinzaparin sodium is a LMWH, deriving from UFH via enzymatic depolymerization. Due to its specific way of preparation, it presents several unique pharmacokinetic and pharmacodynamic characteristics making its once daily administration both efficacious and safe. Because of its higher MW (an average of 6500 Da) compared to other LMWHs, it is eliminated in both saturable and non-saturable way, thus having a favorable profile for specific populations such as elders or patients with renal insufficiency. There is evidence supporting its use in obese patients and during pregnancy as well. It also presents anti-inflammatory and anti-oncogenic action mediated mainly via the TFPI pathway. Further studies will elucidate its clinical utility on immunothrombosis treatment in context of cancer and infections.

## Data Availability

Not applicable.
